# The MIS-HF in clinical practice

**DOI:** 10.1007/s12471-022-01731-6

**Published:** 2022-11-10

**Authors:** A. J. Gingele, J. Boyne, C. Knackstedt, H. P. Brunner-La Rocca

**Affiliations:** 1 Department of Cardiology, Maastricht University Medical Centre+, Maastricht, The Netherlands; 2 Department of Patient and Care, Maastricht University Medical Centre+, Maastricht, The Netherlands; 3 Department of Health Services Research, CAPHRI, Maastricht University, Maastricht, The Netherlands

Dear editor,

Recently, we published the results of the Maastricht Instability Score—Heart Failure (MIS-HF) trial [1]. We concluded that the MIS-HF questionnaire successfully identified stable heart failure patients in secondary care who could safely be referred to primary care. So we read with great interest the commentary by Meregalli regarding our publication in the Netherlands Heart Journal [2].

The heart failure epidemic is a serious threat to health-care systems, and appropriate risk stratification of heart failure patients may help for optimal resource utilisation. Numerous risk scores have been developed to support treatment decisions in heart failure care. Still, because the risk scores are not related to clinical decision-making, hardly any are used in clinical practice. MIS-HF is an exception in this respect and has actually been implemented in clinical practice.

We fully support Meregalli’s suggestion that MIS-HF should also be implemented in primary care. In fact, we tried to recruit general physicians to use the recommendations from MIS-HF for the referral of heart failure patients. Unfortunately, most GPs refused to participate, possibly—and also suggested by Meregalli—because the use of laboratory and ECG data can make it rather complex. It remains to be investigated whether the use of a two-step procedure with an initial simple screening, as suggested, is equally effective and safe.

Meregalli questions whether patients with New York Heart Association (NYHA) class III could be considered stable, as those patients were not excluded from referral to primary care. Still, symptoms are subjective experiences and differentiating between NYHA class II or III can be challenging in clinical practice. Although we are aware of the poor prognosis of patients with significant symptoms, it can be argued that patients with unstable heart failure do not have NYHA III dyspnoea without any other symptoms or other signs of instability. Thus, scoring of NYHA class may not be seen in isolation. This is supported by our results, as patients classified as stable by MIS-HF had a significantly better prognosis than patients with higher scores.

One further word of caution was mentioned regarding the 6% of patients classified as stable who died within one year of follow-up, suggesting suboptimal discriminating power of MIS-HF. We do agree that HF is characterised by high mortality and frequent hospitalisations, even when patients are stable. However, the question arises as to whether prognosis can be improved in these patients when they remain in secondary care. Our results suggest that this is not the case, which is in agreement with the NORTHSTAR trial [3]. Given the fact that we included an elderly population with a mean age of 74 years, patients may also die from other causes than heart failure. Nevertheless, and as proposed by Meregalli, patients from our study who were referred to primary care could easily re-access secondary care if and when needed, highlighting the need of good communication between primary and secondary care.

Lastly, bridging the gap between risk assessment and clinical decision-making is urgently warranted. This, however, requires testing of clinical implications of risk prediction. As in MIS-HF, this can be done in a retrospective cohort study. Thereafter, the instrument needs to be validated in a prospective intervention trial.
